# 222. Oral Step-Down Therapy for Uncomplicated *Enterococcus* Bacteremia

**DOI:** 10.1093/ofid/ofad500.295

**Published:** 2023-11-27

**Authors:** Jessica Ni, Arnold Decano, ioannis Zacharioudakis, Dana Mazo, Yanina Dubrovskaya, Justin Siegfried, Kassandra Marsh

**Affiliations:** NYU Langone Hospital - Brooklyn, San Jose, California; NYU Langone Health, New York City, New York; New York University, New York city, New York; New York University, New York city, New York; NYU Langone Health, New York City, New York; NYU Langone Health, New York City, New York; NYU Langone Health, New York City, New York

## Abstract

**Background:**

The mainstay of therapy for uncomplicated Enterococcal bloodstream infections (BSI) is intravenous (IV) antibiotics which have higher bioavailability than oral (PO) options. This study evaluated clinical outcomes and the safety of early step-down to oral antibiotics in uncomplicated *Enterococcus* bacteremia.

**Methods:**

This was a retrospective study of adult patients admitted from January 2013 to October 2022 with an initial bacteremia episode and positive blood culture for *Enterococcus* species who completed treatment with PO or IV antimicrobial therapy. The primary endpoint was clinical cure, defined as no evidence of transition back to IV therapy after starting PO therapy or no need for escalation of definitive IV therapy due to new fever, leukocytosis, or hemodynamic instability. Safety endpoints were *C. difficile* or adverse drug events leading to discontinuation or change in therapy.

**Results:**

A total of 110 patients were included in the analysis, and 49% received oral step-down therapy (n=54). The most commonly used PO agents were linezolid (59%) and amoxicillin (22%). The severity of illness of the IV and PO groups was assessed by Pitt bacteremia score [1.7 (interquartile range, 0-2.8) vs. 1.3 (IQR, 0-2.0)], Charleston Comorbidity Index [6 (IQR, 4.0-8.0) vs. 5 (IQR, 3.8-6.0)] and ICU admission (35.2% vs. 13.0%; P=0.008). The most commonly identified sources of infection were gastrointestinal (23%) and urinary (23%). No statistical differences were seen between the IV and PO groups in clinical cure (91.1% vs. 98.1%; P=0.111), microbiological cure (98.2% vs. 100%; P=0.509), mortality (1.9% vs. 8.9%; P=0.111), or infectious readmissions at 30-day (5.6% vs. 12.5%; P=0.163) and 90-day (14.8% vs. 19.6%; P=0.384). HLOS was 12.5 days in the IV group and 8.5 days in the PO group (P=0.038). No differences were seen in safety outcomes. The majority of patients in both the IV and PO groups were followed by ID consult (76.8% vs. 81.5%; P=0.356) and Antimicrobial Stewardship (75.0% vs. 77.8%; P=0.453).

**Conclusion:**

Our results suggest that oral antibiotic step-down therapy in uncomplicated Enterococcal BSIs may be a safe alternative to IV therapy in a carefully selected patient population. Larger studies are required to confirm these results.

**Disclosures:**

**All Authors**: No reported disclosures

